# GammaTile^®^ Brachytherapy as Spatially Fractionated Radiation Therapy for Postoperative Brain Cancer: A Paradigm for Precision Intracranial Radiotherapy

**DOI:** 10.3390/cancers18193201

**Published:** 2026-10-03

**Authors:** Hualin Zhang, Nguyen Phuong Dang, Jason C. Ye, Adam Garsa, Aram S. Modrek, Zhilei Liu Shen, Salim Balik, Zhengzheng Xu, Kaley Woods, Adam C. Turner, David G. Brachman, Thomas Chen, Gabriel Zada, Eric L. Chang

**Affiliations:** 1 Department of Radiation Oncology, University of Southern California, Los Angeles, CA 90033, USA; 2 Department of Medical Affairs, GT Medical Technologies, Inc., Tempe, AZ 85281, USA; 3 Department of Neurological Surgery, University of Southern California, Los Angeles, CA 90033, USA

**Keywords:** GammaTile brachytherapy, spatially fractionated radiation therapy, high dose core number density

## Abstract

**Purpose:** Tile-based radiation therapy (TBRT) delivers adjuvant radiation to brain tumor beds using evenly spaced cesium-131 (Cs-131) sources in collagen tiles. This study evaluated TBRT as spatially fractionated radiation therapy (SFRT) across tumor sizes and source counts. **Methods and Materials:** Dose distributions were generated from postoperative CT scans for 18 patients treated with TBRT, using 10–32 implanted Cs-131 sources per case (median, 20). The resection cavity (RC) was contoured on CT with registered postoperative T1-weighted MRI guidance. CTV_5mm was defined as the RC plus a 5 mm expansion excluding skull and surgical tract, and CTV_5mm_Eval as CTV_5mm minus the RC. Both volumes were analyzed using SFRT dose metrics from the Radiosurgery Society SFRT/Flash working group white paper, and planar high-dose core number density (HCND_A) was used to characterize TBRT spatial fractionation. **Results**: The mean dose indices of D5 (dose covering 5% of the target volume), D10, D50, D90, D95 and D100 were 175.8 ± 43.3, 148.8 ± 35.1, 86.7 ± 18.5, 56.1 ± 10.5, 50.2 ± 9.0 and 33.4 ± 7.2 Gy for the CTV_5mm_Eval, respectively. The heterogeneity indices, represented by the ratio of D5/D95 and D10/D90, were 3.5 ± 0.6 and 2.7 ± 0.4, respectively, which were in the range of the linac-based Lattice therapy. HCND_A was a constant, TBRT delivered one high dose core for every 1 cm^2^ of tumor bed. All dose metrics in both the CTV_5mm and CTV_5mm_Eval were largely consistent across all plans. **Conclusions**: TBRT produced heterogeneous dose distributions comparable to conventional SFRT. A 60 Gy TBRT nominal prescription dose approximated the SFRT D90, or valley dose of the 5 mm expansion target volume, suggesting TBRT may provide an SFRT platform for tile-accessible cancers.

## Simple Summary

Tile-based radiation therapy (TBRT) delivers postoperative radiation to brain tumor beds using evenly spaced cesium-131 sources embedded in collagen tiles. This study examined TBRT as a form of spatially fractionated radiation therapy (SFRT) across tumors of different sizes and varying tile/source counts. Although SFRT is increasingly used clinically, the optimal prescription dose remains debated. In GRID therapy, peak dose has traditionally guided prescription, but recent preclinical evidence suggests that valley dose may also be important. Analysis of 18 TBRT cases showed that GammaTile offers a postoperative SFRT approach for brain tumor treatment that is comparable to GRID therapy. GammaTile-based SFRT achieved consistent dose heterogeneity and high-dose core density across plans, similar to linac-based SFRT. The 60 Gy dose used in TBRT corresponds to the dose covering 90% of target volume, and TBRT’s reproducibility and ease of implementation may support selected clinical applications and multi-institutional SFRT trials.

## 1. Introduction

Radiation therapy is a standard component of adjuvant treatment following resection for certain types of operable brain tumors [1,2,3]. Traditionally, adjuvant radiation is delivered using conventional or hypofractionated external beam radiation therapy (EBRT), depending on the tumor histology. A common clinical challenge is managing the time in between resection and initiation of irradiation that is required for surgical recovery and EBRT treatment planning. Residual tumor progression and rapid recurrence may occur during this treatment gap and longer duration between resection and EBRT is correlated with worse outcomes [4,5].

**Figure 1:**
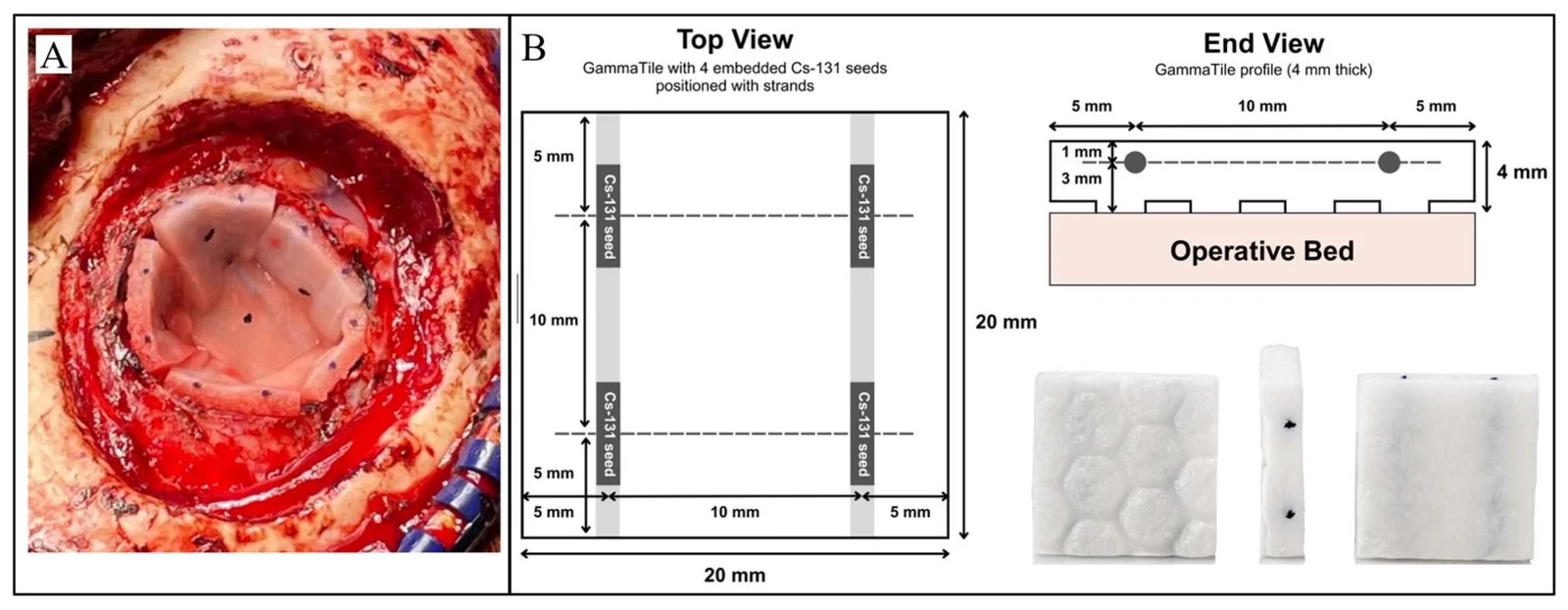
(**A**) Tile-based radiation therapy implant in a brain metastasis resection cavity. (**B**) Engineering and photography of a GammaTile with embedded four Cs-131 sources. Images provided by GT Medical Technologies Inc. Abbreviations: Cs-131, cesium-131.

Recently, tile-based radiation therapy (TBRT) has emerged as an alternative to EBRT. TBRT is performed by lining at-risk surfaces of a surgical bed with multiple GammaTile^®^ (GT Medical Technologies Inc., Tempe, AZ, USA) devices following maximal safe brain tumor resection (Figure 1A). TBRT ensures that delivery of radiation dose begins immediately at time of surgery and avoids the issues associated with delayed adjuvant radiation treatment or access to care. A GammaTile device is a low dose rate brachytherapy applicator that is FDA cleared for intracranial neoplasms. Each device consists of a 2 cm × 2 cm × 0.4 cm bioresorbable collagen tile embedded with four Cs-131 sealed sources (seeds) [6,7], each with a nominal implanted air kerma strength of 3.5 cGy cm^2^ hr^−1^ (Figure 1B). The design of the GammaTile ensures a uniform distribution of seeds with a spacing of approximately 1 cm that remains constant throughout the course of TBRT treatment.

Several single-arm prospective and retrospective TBRT studies have reported favorable local control (LC) and radiation necrosis (RN) outcomes relative to those reported for adjuvant EBRT for multiple brain tumor histologies, including glioblastoma, meningioma, and brain metastases (BM) [8,9,10]. Notably, several of these studies demonstrated that TBRT is effective and safe for the treatment of previously irradiated resection cavities that locally recurred following one or more courses of EBRT [9,11,12,13,14]. These findings suggest that TBRT may offer dosimetric or biologically based therapeutic advantages for brain tumors with intrinsic or acquired radioresistance, as seen in some glioblastomas [2,3], meningiomas [3], and BM [15].

Managing radioresistant tumors remains a formidable challenge despite ongoing advances in radiation delivery and systemic therapies [16]. Radioresistance refers to the capacity of tumor cells to survive and proliferate despite therapeutic radiation. It is typically driven by tumor heterogeneity, specific genetic mutations, and adaptive changes in the tumor microenvironment [17,18,19]. In addition to surgical resection and integration of systemic therapies, treatment strategies for radioresistant tumors include radiation dose escalation, intensification, or altered fractionation; however, these approaches are frequently constrained by normal tissue tolerance and may increase treatment-related toxicities.

Spatially fractionated radiation therapy (SFRT), the unique method of treating tumors by delivering intentionally designed heterogeneous dose distributions, is an area of increasing interest in radiation oncology, physics, and tumor biology [20]. SFRT started with GRID therapy using GRID block collimators, in which a uniform radiation field was converted into multiple beamlets in a fixed pattern [21]. Advanced treatment planning systems (TPS) and on-board multileaf collimator (MLC) enabled a second type of SFRT, lattice radiation therapy (LRT), which delivers three-dimensionally populated high dose islands inside the tumor volume. Clinical experience with SFRT suggests higher response rates and lower toxicity compared with unform dose delivery in the treatment of conventional therapy-refractory and advanced tumors [22,23]. SFRT clinical data are rapidly accumulating while the applications and techniques are quickly evolving, as the mechanisms of SFRT are an ongoing area of research [24].

**Figure 2:**
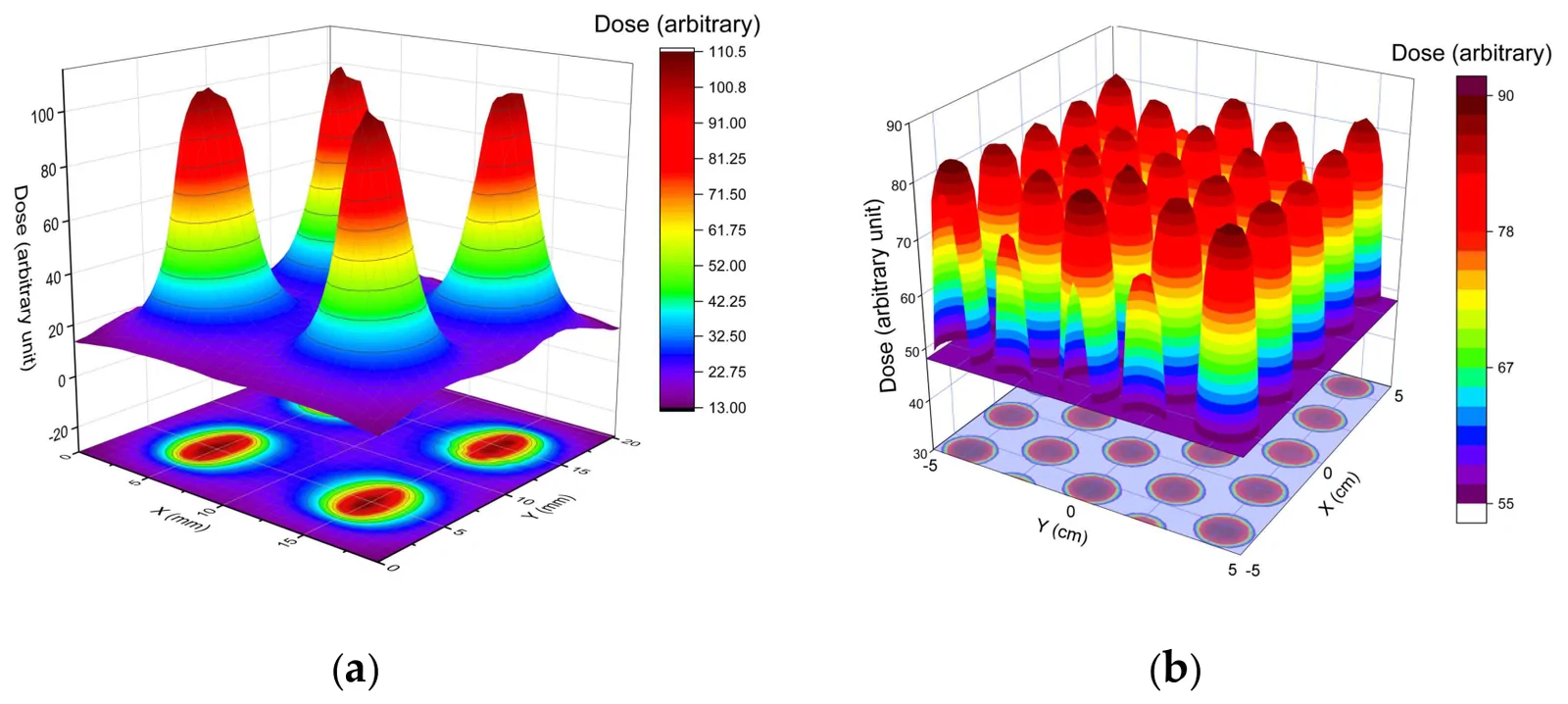
Film dosimetry of a single GammaTile (**a**) and GRID therapy (**b**).

It is notable that the favorable LC and RN rates reported with TBRT for recurrent, potentially radioresistant brain tumors, relative to outcomes with conventional EBRT, are qualitatively similar to the improvements reported with SFRT compared with conventional EBRT for radioresistant intact tumors [22]. Furthermore, due to the GammaTile design, which ensures regular-separation seed distribution, and the low-energy (~30 keV) photons emitted by Cs-131, the dose distribution to tissue adjacent to the surgical bed is expected to be highly heterogeneous, similar to that observed with SFRT. For qualitative comparison of dose heterogeneity and spatial fractionation, Figure 2 presents a film-based dose measurement for a single 2 cm × 2 cm GammaTile alongside a representative GRID therapy plan. For the GammaTile dose measurement, the film was placed on a plane 3 mm from the source, whereas for the GRID dose measurement, the film was positioned at depth of maximum dose for a 6-MV beam (1.5 cm).

The purpose of this work is to retrospectively evaluate the heterogeneity of patient-specific dose distributions from TBRT implants to target volumes in terms of commonly utilized SFRT dosimetry metrics. A comparison demonstrating similar heterogeneity characteristics may suggest that the mechanistic principles proposed for SFRT in the treatment of radioresistant tumors are also applicable to TBRT, thereby providing further insight into its effectiveness for adjuvant radiation treatment of resected radioresistant brain tumors.

## 2. Methods and Materials

A total of 18 consecutive patients with BMs treated at a single center who underwent surgical resection and TBRT were retrospectively analyzed for this dosimetry study. In this patient cohort, there were a total of 18 resected BM cavities. The number of GammaTiles (Tempe, AZ, USA) ordered for each patient was determined using a preoperative, diagnostic, T1-weighted, contrast-enhanced magnetic resonance imaging series (T1+C MRI) by either delineating the preoperative BM volume and estimating the anticipated surgical bed surface area requiring treatment or by using a vendor-supplied look up table that uses the maximum tumor dimension. Immediately following maximal safe tumor resection, the appropriate number of GammaTiles were placed by a neurosurgeon along at-risk surfaces of the surgical bed, without gaps or overlap between adjacent tiles and with each tile oriented to maintain a 3 mm offset between the seed plane and the cavity surface (i.e., textured side in contact with tissue). When necessary, tiles were cut in half to accommodate the size and shape of surgical bed. The number of implanted GammaTiles across cases ranged from 2.5 to 8, corresponding to 10 to 32 implanted seeds.

**Figure 3:**
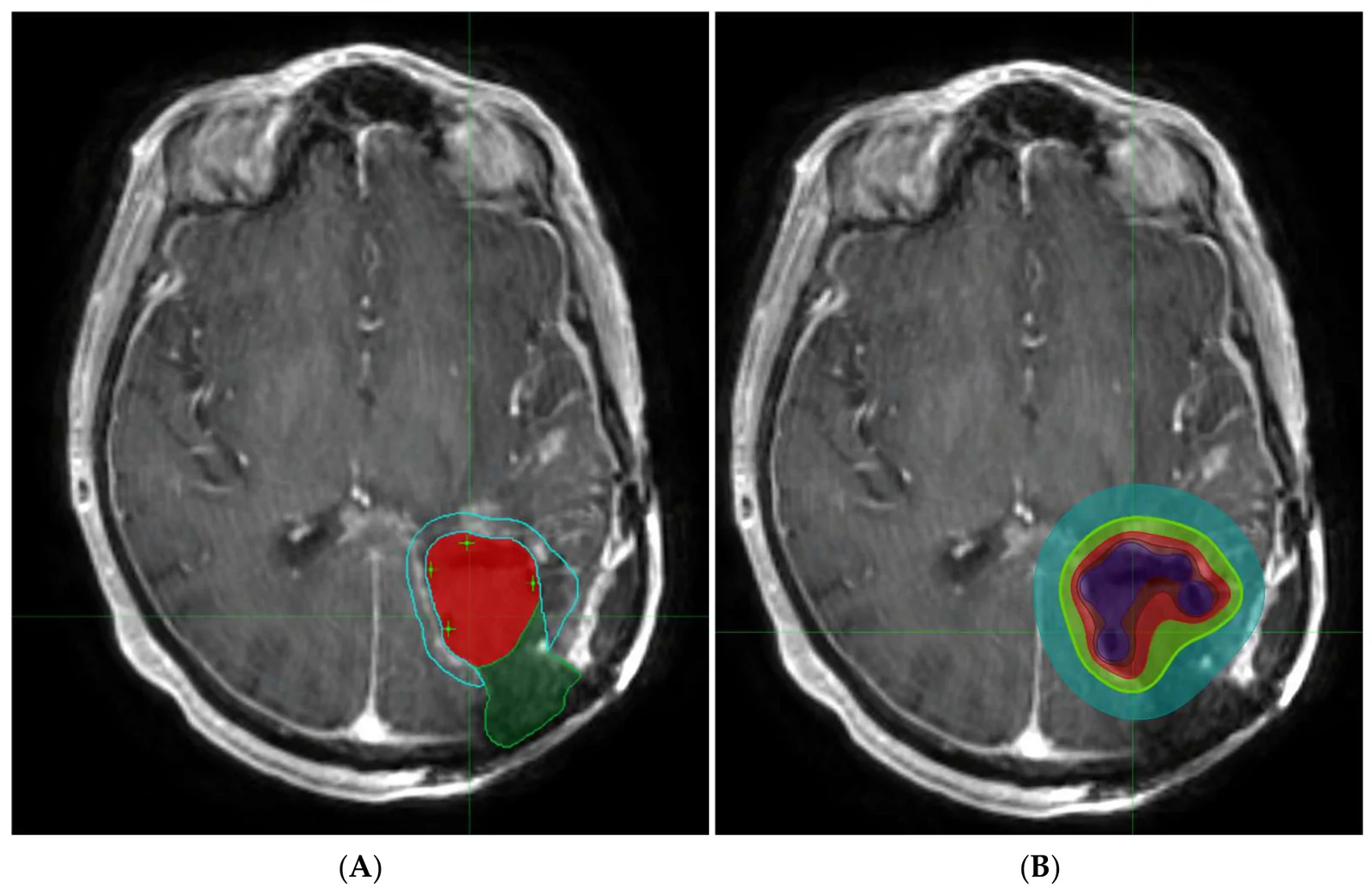
Axial T1+C MRI displaying: (**A**) visible Cs-131 source positions (green “+”) and contoured structures, including the RC (shaded red), surgical pathway (shaded dark green), CTV_5mm_Eval (cyan). Note, the CTV_5mm is the union of the RC and CTV_5mm_Eval. (**B**) TBRT isodose distribution (image key includes dose values, in Gy, corresponding to shaded volumes). The green line represents 60 Gy. Abbreviations: RC, resection cavity; TBRT, tile-based radiation therapy.

For each patient, a postoperative computed tomography (CT) scan (with a predefined CT scanner protocol consisting of a non-overlapping 2 mm slice helical scan, with metal artifact reduction automatically applied) and an MRI exam (1 mm slice thickness) were obtained within 24 h of resection and TBRT implant. The CT scan and the T1+C MRI sequence were fused using rigid registration using MIM^®^ (Version 7.3.2, MIM Software Inc., Cleveland, OH, USA). When same-day post-implant CT and MRI head images are imported into MIM, MIM performs an automatic anatomic structure registration, and a physicist then verifies the registration quality slice by slice to make sure the registered images are satisfactory. Then, the entire surgical pathway and the sub-portion deemed to represent the high-risk resection cavity (RC), as indicated by visible seed placement, were separately contoured on the CT by a medical physicist, with the fused T1+C MRI used for guidance when necessary. Next, the clinical target volume (CTV_5mm) was generated by expanding the RC by 5 mm and manually modifying to remove overlap with uninvolved tissue (i.e., skull and surgical pathway outside the RC). A 5 mm CTV expansion margin was selected to align with the commonly used TBRT dose-depth specification of 60 Gy at 5 mm and the target volume definitions used in prior TBRT dosimetric studies [25]. A second target volume (CTV_5mm_Eval) was generated by subtracting the RC from the CTV_5mm to exclude regions not expected to contain residual tumor cells (see Figure 3A). Finally, each seed position was localized, and the TBRT dose distribution (see Figure 3B) was calculated using MIM Symphony^®^ (Version 7.3.2, MIM Software Inc., Cleveland, OH, USA), which implements the American Association of Physicists in Medicine Task Group 43 formalism with a per source air kerma strength of 3.5 cGy cm^2^ hr^−1^ [26,27]. The seed positions were independently verified by a second physicist.

In the context of SFRT, the dose metrics recommended by the Radiosurgery Society (RSS) GRID/Lattice/Flash Physics Working Group white paper [28] were extracted from the retrospectively obtained TBRT plans. The doses covering 100% (D100), 95% (D95), 90% (D90), 50% (D50), 10% (D10), and 5% (D5) of the CTV_5mm and CTV_5mm_Eval volumes (denoted dose–volume coverage) were obtained for all cases. The values of the dose–volume coverage metrics were used to calculate heterogeneity indices, consisting of D5/D95 and D10/D90, which serve as surrogates for peak-to-valley dose ratios (PVDR) in complex SFRT plans. Thus, the use of arbitrary peak and valley doses could be avoided.

In order to evaluate the SFRT-like periodic and often archipelago-like dose distribution from TBRT, the high dose core number density (HCND) was calculated. Since the unform source distribution for TBRT is similar to a collimator-based or MLC-based GRID therapy that provides 2D dose modulation and the target volume is relatively planar with respect to the GammaTile device, the HCND of cross-sectional area (HCND_A) was utilized for this work [25,26]. HCND_A is calculated as:

(1) $$HCND\_A=\frac{n}{A}\times \;100\;cm^{2}$$

where *n* is the number of vertices or high dose cores (equivalent to number of seeds in the TBRT implant) and *A* is the target cross section (equivalent to the surface area of the TBRT implant). In GammaTile implant practice, the tiles are placed on the tumor bed without overlapping; therefore, we use the area of tiles to represent the tumor bed surface area. HCND_A effectively indicates the number of high dose cores for every 100 cm^2^.

## 3. Results

### 3.1. Dose Volume Histogram of TBRT Cases

**Figure 4:**
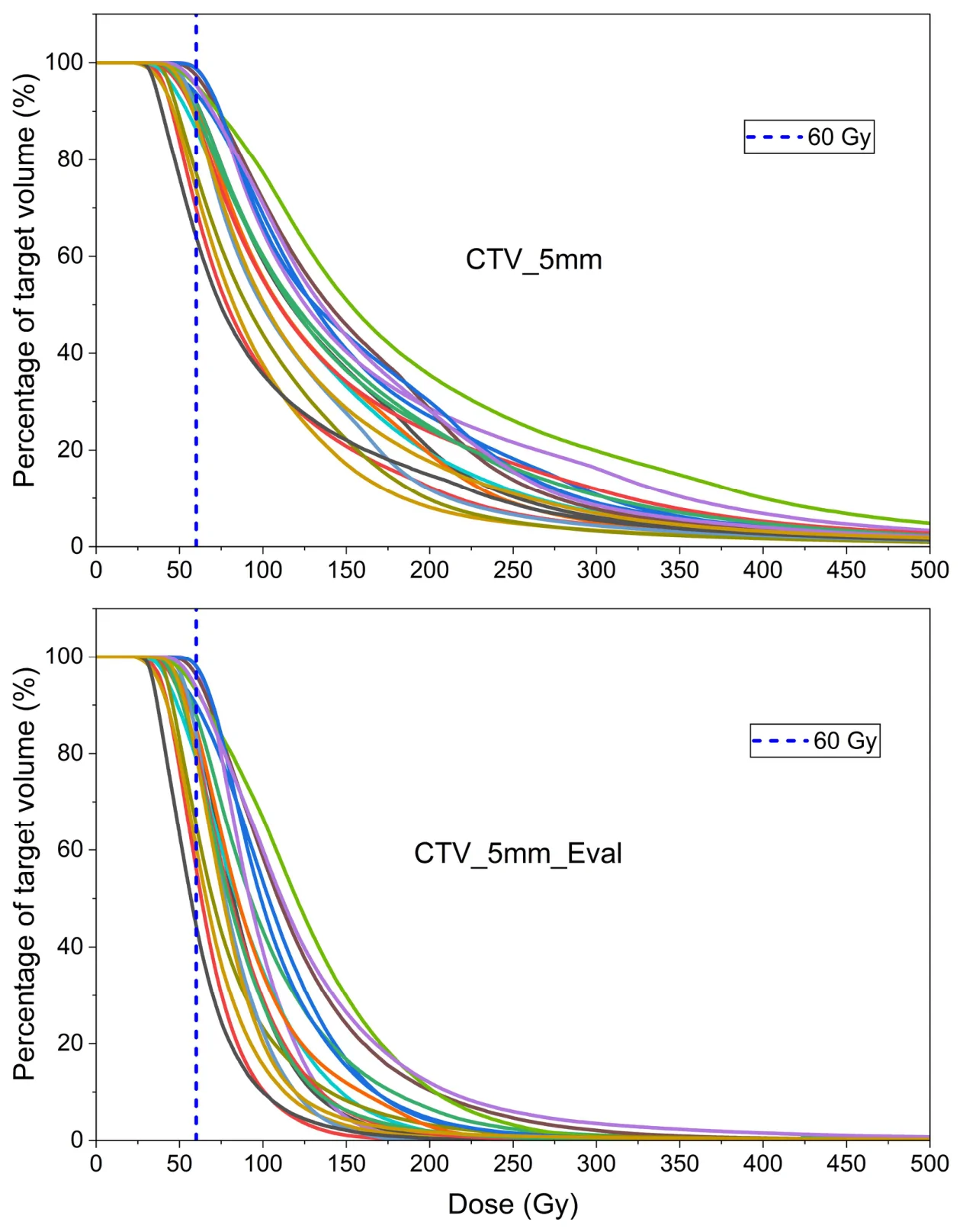
Dose volume histograms for the CTV_5mm and CTV_5mm_Eval target volumes for all patients. Different colors represent different cases.

Dose–volume histograms (DVH) for the CTV_5mm and CTV_5mm_Eval are shown in Figure 4 and summarized in Table 1 and Table 2 for all 18 TBRT cases. DVH curves demonstrated broadly consistent patterns across patients even with varying RC volumes, numbers of implanted sources, and anatomical locations. Similar levels of consistency and variation have been reported in linac-based GRID and LRT [29,30].

### 3.2. SFRT Dose Metrics

**Figure 5:**
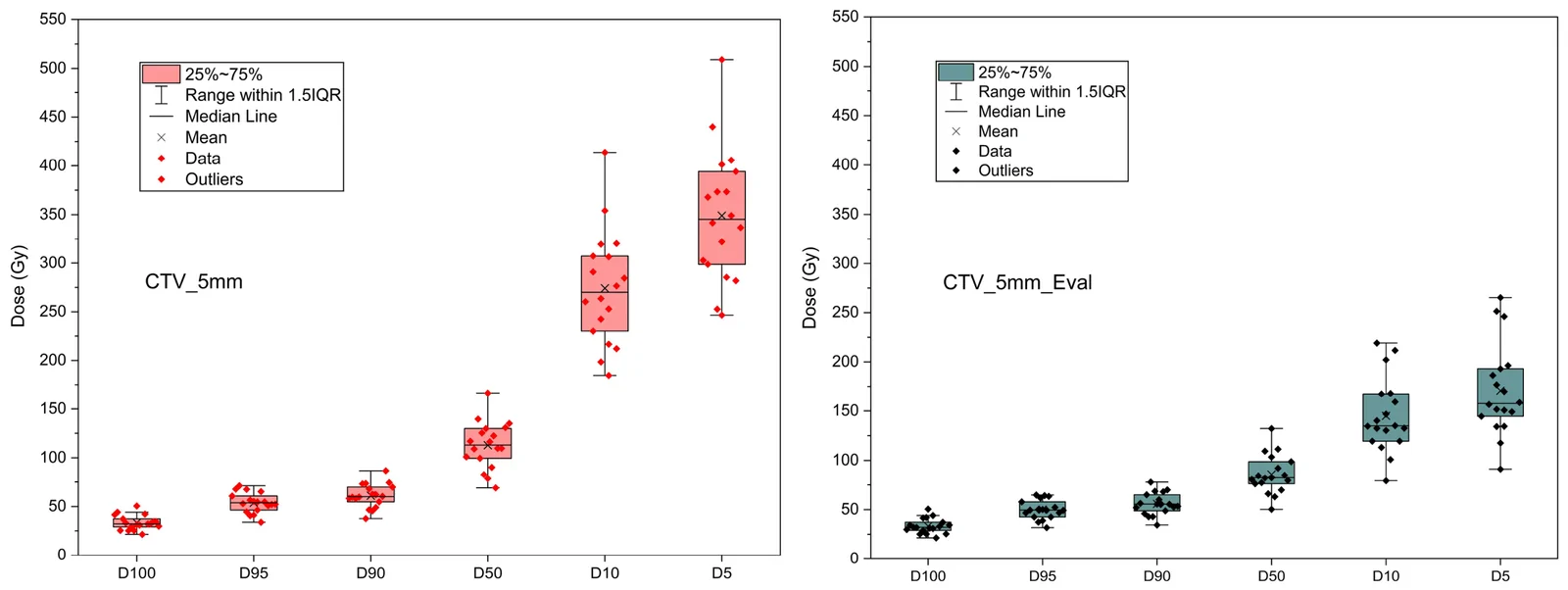
Box-and-whisker plots of dose–volume coverage metrics (D100, D95, D90, D50, D10, and D5) for all patients for the (**left**): CTV_5mm and (**right**): CTV_5mm_Eval target volumes. Boxes represent the IQR, with the median shown as a horizontal line and the mean indicated by an “x”; whiskers extend to 1.5 × IQR, and points denote individual patient values. Abbreviations: Dx, dose covering x% of target volume (Gy); IQR, interquartile range.

SFRT dose metrics derived from the dose volume histograms for all patients and summary statistics, including the mean, standard deviation, and percent standard deviation across patients, are presented in Table 1 and Table 2 for the CTV_5mm and the CTV_5mm_Eval, respectively. Figure 5 shows box-and-whisker plots of dose–volume coverage values for both target structures for visual comparison. The magnitude of the dose–volume coverage variation across patients increased as the volume threshold value decreased; however, the percent standard deviation remained relatively consistent.

### 3.3. Dose Heterogeneity Metrics

**Figure 6:**
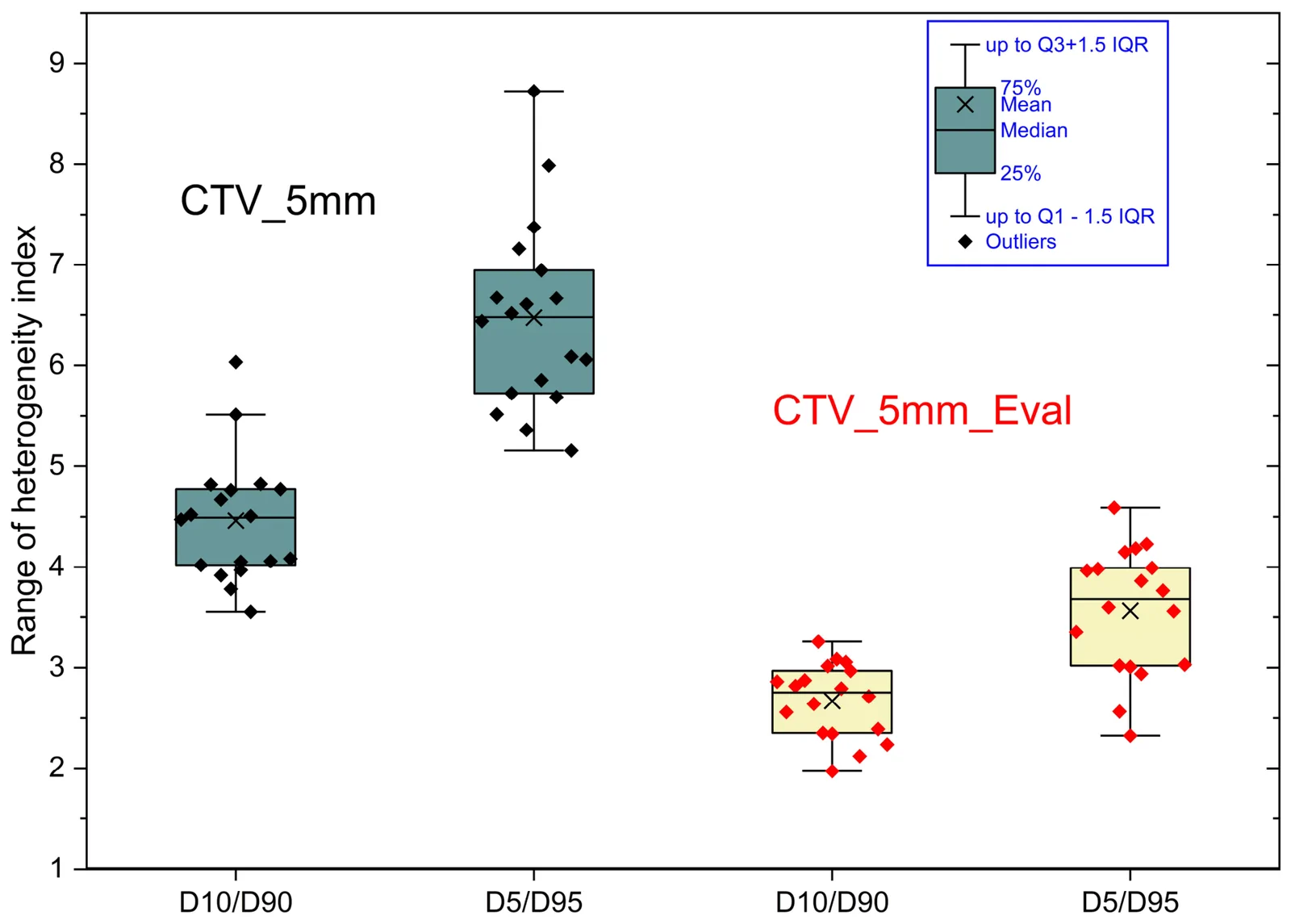
Box-and-whisker plots of heterogeneity indices (D10/D90 and D5/D95) for all patients for the CTV_5mm (blue) and CTV_5mm_Eval (yellow) target volumes. Boxes represent the IQR, with the median shown as a horizontal line and the mean indicated by an “x”; whiskers extend to 1.5 × IQR, and points denote individual patient values. Abbreviations: IQR, interquartile range; Q1, first quartile (25th percentile); Q3, third quartile (75th percentile).

The values and summary statistics for dose heterogeneity indices, including D10/D90 and D5/D95, are presented for the CTV_5mm and CTV_5mm_Eval in Table 1 and Table 2. This data is graphically illustrated using box-and-whisker plots for both target volumes in Figure 6. The mean values of D10/D90 and D5/D95 across patients for the CTV_5mm were 4.5 ± 0.6 and 6.5 ± 0.9, respectively. Similarly, for the CTV_5mm_Eval, the mean D10/D90 and D5/D95 were 2.7 ± 0.4 and 3.5 ± 0.6. The percent standard deviation for the D10/D90 and D5/D95 was lower for the CTV_5mm (14% and 14%, respectively) than for the CTV_5mm_Eval (13% and 17%, respectively), indicating slightly reduced relative interpatient variability in heterogeneity indices when the RC is included in the CTV structure.

### 3.4. HCND_A Metrics

HCND_A for TBRT is calculated using Equation (1) while noting *n* is the number of implanted tiles multiplied by 4 seeds per tile and *A* is the number of implanted tiles multiplied by the surface area of each tile (4 cm^2^). So, for any TBRT implant:

(2) $$HCND\_A=\frac{n}{A}\;\times \;100\;cm^{2}=\frac{tiles\;\times \;4\;seeds/tile}{tiles\;\times \;4\;cm^{2}/tile}\times \;100\;cm^{2}=100$$

An HCND_A of 100 indicates there are 100 high dose cores per 100 cm^2^, or equivalently, 1 high dose core per 1 cm^2^. This suggests that, as long as there is no gap or overlap, TBRT maintains the same high-dose core density across all plans.

## 4. Discussion

When used for adjuvant treatment following resection of operable brain tumors, TBRT initiates the delivery of radiation dose immediately at the time of surgery via multiple Cs-131 seeds embedded within collagen GammaTiles, ensuring a uniform distribution of radiation sources throughout the implant tumor bed. Each source may be conceptualized as an individual radiation beamlet, analogous to those used for SFRT, resulting in a non-uniform dose distribution to tissue adjacent to the RC. Given this expected heterogenous dose distribution, this work evaluated dosimetry metrics commonly used to characterize SFRT modalities, such as GRID or LRT, using patient-specific TBRT dose distributions.

The calculation of heterogeneity indices, which serve as surrogates for the PVDR in target volume, relies on dose–volume coverage statistics. The values of coverage dose increased as volume thresholds decreased (i.e., D100 < D95 < … < D5). This trend is expected as TBRT is fundamentally a brachytherapy modality using low-energy Cs-131 sources, which deliver high doses near implanted seeds with steep dose gradients arising from approximate inverse-square dose falloff. In clinical practice, the nominal TBRT dose is commonly specified as 60 Gy to a depth of 5 mm from the RC surface for documentation purposes. Dosimetric evaluation for 18 cases resulted in a mean D90 of 61.1 ±11.5 Gy for the CTV_5mm and 56.1 ±10.5 Gy for the CTV_5mm_Eval. These findings indicate that choosing a dose specification of 60 Gy to a depth of 5 mm is reasonable as a descriptor of the expected D90, or valley dose within the heterogeneous dose distribution; however, variation is expected in routine TBRT practice and depends on the definition of the target volume. This interpretation of valley dose and effectiveness of SFRT is supported by a recent preclinical study showing that valley dose was associated with survival in mice treated with SFRT [31].

This study also assessed the dose–volume coverage variability to indicate whether the reported mean values are broadly representative of this patient cohort. The magnitude of interpatient dose-coverage variability increased as the volume threshold decreased (i.e., the standard deviations of D100 < D95 < … < D5). This trend may be partly attributable to subjectivity inherent in contouring the RC used to generate the CTV_5mm and CTV_5mm_Eval structures. This is because lower volume thresholds (e.g., D5 or D10) sample high-dose regions near the seeds, where dose gradients are steep, and therefore these metrics are highly sensitive to contouring variability. Conversely, higher volume thresholds (e.g., D90 or D100) correspond to lower-dose regions with flatter dose gradients and are less sensitive to contour variability. Despite this trend, the percent standard deviation (i.e., coefficient of variation) remained relatively consistent across all volume thresholds, ranging from 18% to 22% for the CTV_5mm and 18% to 25% for the CTV_5mm_Eval. This consistency suggests that the magnitude of variability across patients increased approximately in proportion to the corresponding dose–volume coverage mean values, supporting the use of these mean values as representative descriptors of TBRT dose distributions in this cohort.

PVDR is an important parameter of SFRT, which represents the degree of dose modulation or heterogeneity. PVDR of GRID therapy decreases with the tumor size and the depth [29,32], so a larger or deeper seated tumor will see a smaller PVDR [33]. This trend was not observed for TBRT, as heterogeneity indices (D10/D90 and D5/D95) remained relatively consistent across all the cases, as evidenced by the relatively low percent standard deviations. Clinically, heterogeneity indices for TBRT are most appropriately evaluated using the CTV_5mm_Eval, as this structure conceptually contains only at-risk tissue and omits the portion of the CTV where residual disease is not expected. For the CTV_5mm_Eval, the D10/D90 values ranged from 2.0 to 3.3 (mean of 2.7). This falls just below the range of D10/D90 reported for collimator-based GRID therapy (3.9–5.0) [29] and within the reported range for linac-based LRT (2.04–5.99) [30]. D5/D95 of TBRT ranged from 2.3 to 4.6 (mean of 3.5), which slightly overlaps the lower end of the reported range for GRID therapy (4.3–7.2) [33] and falls within the lower part of the range for LRT (2.23–11.42) [30]. So, although TBRT shares similarities with GRID therapy, its heterogeneity characteristics are more comparable to those reported for LRT [34].

Since dose–volume coverage based heterogeneity metrics (D10/D90 and D5/D95) do not fully describe the spatial dose modulation essential to SFRT, HCND was used to characterize the SFRT-like spatial dose pattern of TBRT. Zhang et al. [29] introduced two HCND formulations to quantify either volumetric high-dose core density, HCND, or planar high-dose core density, HCND_A, within the target region. HCND_A was considered the more appropriate descriptor for TBRT because this metric was defined to characterize 2D modulated SFRT modalities, such as GRID therapy, which is geometrically analogous to the planar arrangement of evenly spaced Cs-131 sources embedded within the GammaTile device. Furthermore, HCND depends on target volume, which varies for TBRT according to the selected CTV expansion margin around the resection cavity and may therefore differ for a given implant depending on the clinical scenario. In contrast, the planar source density of the TBRT device is fixed by design and so HCND_A may be compared with the source or beamlet spacing used in established SFRT modalities.

For a standard TBRT implant, HCND_A is 100 high-dose cores per 100 cm^2^, or 1 source per cm^2^. Although inherent to the GammaTile design, this fixed density quantitatively supports TBRT as a regularly spaced heterogeneous dose pattern rather than a single peak or irregular high-dose distribution. TBRT therefore demonstrates spatial dose modulation comparable to conventional GRID therapy, with both approaches producing centimeter-scale, regularly spaced high-dose regions. In a recent GRID-therapy-planning study, researchers used a commercially available GRID collimator with 1.0 cm apertures and 1.4 cm center-to-center spacing to evaluate dose metrics and therapeutic ratios across bulky tumors of varying sizes, depths, and anatomical sites [29]. This GRID collimator configuration has been used clinically for decades [22,35]. Although TBRT was not developed specifically as an SFRT technique, its heterogeneous dose pattern exhibits several defining SFRT characteristics, including regularly spaced high-dose islands, elevated PVDRs, and a consistent HCND. These characteristics may help explain the favorable LC and toxicity outcomes reported in clinical practice.

It should be emphasized that the target volumes used for this study to assess dose heterogeneity were generated using a 5 mm expansion from the RC into the surrounding brain parenchyma. The choice of a 5 mm margin was based on prior TBRT studies but remains somewhat arbitrary. As distance from the RC increases, dose heterogeneity decreases due to the rapid dose fall described above. Accordingly, smaller CTV expansion margins will yield higher calculated heterogeneity metrics, whereas larger margins would reduce reported heterogeneity. In fact, PVDRs are maximized in tissue immediately adjacent to the GammaTiles, which also receives the highest overall dose levels and may also pose the heaviest residual cancer cell burden. Therefore, if SFRT-based heterogeneity indices are incorporated into the evaluation of TBRT dose distributions, careful consideration should be given to the choice of expansion margin, guided by patient-specific factors such as extent of resection, RC size and geometry, and tumor histologic characteristics (e.g., diffuse vs. localized disease).

In the postoperative setting, the first few millimeters of brain tissue adjacent to the RC typically contain the highest density of residual tumor cells. This region represents the critical target volume where a high therapeutic ratio is desired; however, because it consists predominantly of normal brain tissue, the safety afforded by spatial fractionation is essential. Our results suggest that TBRT produces high, ablative tumoricidal dose peaks, which are postulated to achieve intensive cell killing alongside relatively low-dose valleys that may help to preserve vascular and immunologic processes. Accordingly, we hypothesize that TBRT and SFRT may share common underlying therapeutic mechanisms, and that the biological advantages proposed for SFRT [20,22] may partially explain the favorable LC and leptomeningeal disease (LMD) outcomes observed for TBRT.

Clinical data to date are consistent with the hypothesis that the SFRT-like dose distribution produced by TBRT may contribute to improved efficacy and safety. In the setting of recurrent BM, reported 1-year LC rates after resection with TBRT range from 83 to 100%, with associated RN rates of 8–16% [9,10,11,12]. These outcomes compare favorably with reported results for recurrent BM treated with repeat stereotactic radiosurgery (SRS) after resection, which have been associated with LC rates of 70–75% [36,37] and symptomatic RN rates of 13% [37,38], respectively. In a recently published randomized, open-label phase 3 trial [39], the resection + TBRT demonstrated both non-inferiority and superiority over the resection + SRS in improving surgical bed recurrence (SBR) and surgical bed recurrence-free survival (SB-RFS) among patients with newly diagnosed brain metastases requiring resection.

These findings should be considered hypothesis-generating, as the strong similarities observed between the absorbed dose distributions produced by TBRT and SFRT do not establish that these dosimetric similarities translate into comparable biological effects. As reported in SFRT, the biological implications of these dose-patterns remain incompletely understood [40]. Future studies should investigate whether the spatially fractionated nature of TBRT produces biological effects analogous to those proposed for SFRT, including differential tumor and normal tissue responses in peak and valley dose regions, preservation of vascular and immune-mediated processes in lower-dose regions, and potential enhancement of tumor control in radioresistant disease. Importantly, the substantially different temporal dose-delivery characteristics of SFRT and TBRT, with SFRT typically delivered in hypo-fractions at high dose rates and TBRT delivered continuously at low dose rates over an extended period, may modify the biological effects associated with spatial dose heterogeneity and should also be investigated. Such work will be necessary to determine whether the observed SFRT-like dosimetric characteristics of TBRT are mechanistically linked to the favorable LC and LMD outcomes reported clinically.

In summary, TBRT produces high ablative dose peaks and relatively low-dose valleys within the postoperative treatment region and appears to do so with consistent dosimetric characteristics across cases. These findings suggest that TBRT is a promising postoperative brain tumor treatment modality with SFRT-like dosimetric characteristics, although future preclinical, translational, and clinical studies are warranted to determine whether these spatially fractionated dose characteristics contribute mechanistically to the efficacy and safety outcomes observed in clinical practice.

**Limitations:** Assessment of TBRT dose to normal tissue outside of the CTV_5mm was not conducted in the current study. Like all forms of radiation therapy, rates of RN or other radiation complications from TBRT are likely associated with the dose to normal tissue outside the target volume, so this should be evaluated in future studies. In addition, CTV expansion margins other than 5 mm were not evaluated in this study; alternative margins may provide different dosimetric insights and should be investigated in future work.

## 5. Conclusions

TBRT delivers spatially fractionated dose distributions with consistent heterogeneity metrics across a range of resection cavity geometries via brachytherapy sources; 60 Gy of TBRT prescription dose was found to approximate the dose covering 90% of target volume, or the valley dose of SFRT. This consistency suggests that these dosimetric characteristics are reproducible and may be reliably evaluated as part of standard TBRT practice or as SFRT clinical trials. The favorable outcomes reported with TBRT in the treatment of previously irradiated, and thus potentially radioresistant, recurrent brain tumors, in terms of both efficacy and safety, may be partly attributable to the high dose heterogeneity and the SFRT profiles observed in this work.

## Figures and Tables

**Table 1:** Summary of implant characteristics and dose–volume metrics for the CTV_5mm. For each patient, the number of implanted seeds, contour volume, and SFRT-dose metrics reported, along with cohort mean, standard deviation, and percent standard deviation were calculated. Abbreviations: Dx, dose covering x% of the CTV_5mm; Std Dev, standard deviation; % Std Dev, coefficient of variation (100 × standard deviation / mean).

Case#	Number of Seeds (n)	CTV_5mm Volume (cm^3^)	D100 (Gy)	D95 (Gy)	D90 (Gy)	D50 (Gy)	D10 (Gy)	D5 (Gy)	D10/D90	D5/D95
1	24	40.7	31.3	55.0	62.4	116.4	252.9	322.0	4.1	5.9
2	16	24.6	25.4	41.1	45.5	78.5	216.7	285.5	4.8	6.9
3	32	47.2	30.9	56.5	68.0	129.9	306.5	373.3	4.5	6.6
4	18	25.2	29.1	54.3	62.2	119.2	290.4	362.0	4.7	6.7
5	20	26.0	50.5	67.5	73.4	125.4	354.1	439.9	4.8	6.5
6	16	27.6	21.5	40.5	46.5	82.6	184.6	246.5	4.0	6.1
7	16	19.4	25.3	46.3	54.7	109.7	263.5	341.5	4.8	7.4
8	24	34.4	42.1	65.1	73.1	139.6	276.5	348.9	3.8	5.4
9	24	55.3	33.1	44.4	48.9	90.2	198.5	252.6	4.1	5.7
10	26	50.2	32.2	52.9	60.3	109.7	242.4	302.9	4.0	5.7
11	20	30.3	37.3	54.7	59.7	99.5	212.1	281.9	3.6	5.2
12	28	29.0	32.3	71.1	86.7	166.4	413.8	509.0	4.8	7.2
13	10	17.9	25.5	35.6	39.5	74.4	238.3	310.6	6.0	8.7
14	16	22.8	34.3	50.8	58.1	109.1	320.2	405.8	5.5	8.0
15	16	17.8	44.1	67.7	74.3	130.9	291.0	373.4	3.9	5.5
16	22	33.5	34.3	52.0	59.4	117.0	307.3	394.2	5.2	7.6
17	14	16.2	41.5	60.7	69.7	135.1	284.6	367.8	4.1	6.1
18	24	42.0	29.9	52.3	58.2	101.0	260.3	336.7	4.5	6.4
Average	20.3	31.1	33.4	53.8	61.1	113.0	273.0	347.5	4.5	6.5
Std Dev	5.4	11.4	7.2	9.7	11.5	22.8	55.1	64.0	0.6	0.9
%Std Dev	27%	37%	22%	18%	19%	20%	20%	18%	14%	14%

**Table 2:** Summary of implant characteristics and dose–volume metrics for the CTV_5mm_Eval. For each patient, the number of implanted seeds, contour volume, and SFRT-dose metrics reported, along with cohort mean, standard deviation, and percent standard deviation were calculated. Abbreviations: Dx, dose covering x% of the CTV_5mm_Eval; Std Dev, standard deviation; % Std Dev, coefficient of variation (100 × standard deviation / mean).

Case#	Number of Seeds (n)	CTV_5mm_Eval Volume (cm^3^)	D100 (Gy)	D95 (Gy)	D90 (Gy)	D50 (Gy)	D10 (Gy)	D5 (Gy)	D10/D90	D5/D95
1	24	21.7	31.3	50.1	55.6	82.2	130.3	150.7	2.3	3.0
2	16	12.5	25.4	38.9	42.9	63.0	100.9	117.5	2.4	3.0
3	32	29.8	30.9	50.0	60.1	103.4	167.9	193.1	2.8	3.9
4	18	17.4	29.1	50.8	57.7	92.5	177.9	212.7	3.1	4.2
5	20	15.4	50.5	64.1	68.5	92.0	135.1	149.1	2.0	2.3
6	16	18.0	21.5	37.4	42.8	66.0	113.1	134.5	2.6	3.6
7	16	12.8	25.3	42.6	48.7	84.3	146.9	170.0	3.0	4.0
8	24	23.2	42.1	61.8	68.1	109.3	202.2	246.1	3.0	4.0
9	24	36.2	33.1	42.6	45.9	69.8	140.2	176.8	3.1	4.2
10	26	32.8	32.1	49.5	55.4	84.9	159.2	186.4	2.9	3.8
11	20	19.5	37.3	52.3	56.3	77.3	119.5	134.2	2.1	2.6
12	28	19.4	32.3	63.4	77.8	132.2	219.2	251.4	2.8	4.0
13	10	11.6	25.5	34.0	36.8	56.8	99.9	121.0	2.7	3.6
14	16	13.7	34.5	46.7	52.6	80.4	134.6	156.6	2.6	3.4
15	16	11.6	44.1	64.8	70.0	98.8	167.4	196.3	2.4	3.0
16	22	16.8	34.5	46.9	52.0	79.5	132.6	158.6	2.6	3.4
17	14	11.7	41.5	57.8	65.0	111.4	211.7	265.3	3.3	4.6
18	24	24.5	29.9	49.2	53.4	76.1	119.5	144.6	2.2	2.9
Average	20.3	19.4	33.4	50.2	56.1	86.7	148.8	175.8	2.7	3.5
Std Dev	5.4	7.3	7.2	9.0	10.5	18.5	35.1	43.3	0.4	0.6
%Std Dev	27%	37%	22%	18%	19%	21%	24%	25%	13%	17%

## Data Availability

The deidentified dosimetry data is available under request to the corresponding author (H.Z.).

## Glossary

BM	Brain Metastases
CT	Computed tomography
CTV	Clinical target volume
CTV_5mm	CTV expanded by 5mm,
EBRT	External beam radiation therapy
HCND	High-dose core number density
HCND_A	High-dose core number density based on the treatment area
LRT	Lattice radiation therapy,
LC	Local control,
RN	Radiation Necrosis
LMD	Leptomeningeal Disease
MRI	Magnetic resonance imaging
PVDR	Peak–valley dose ratio
RC	Resection Cavity
SFRT	Spatially fractionated radiation therapy
SRS	Stereotactic radiosurgery
TBRT	Tile-based radiation therapy
